# Genetic determinants in head and neck squamous cell carcinoma and their influence on global personalized medicine

**DOI:** 10.18632/genesandcancer.110

**Published:** 2016-05

**Authors:** Nicole L. Michmerhuizen, Andrew C. Birkeland, Carol R. Bradford, J. Chad Brenner

**Affiliations:** ^1^ Department of Otolaryngology – Head and Neck Surgery, University of Michigan Medical School, Ann Arbor, MI, USA; ^2^ Department of Pharmacology, University of Michigan Medical School, Ann Arbor, MI, USA; ^3^ Comprehensive Cancer Center, University of Michigan Medical School, Ann Arbor, MI, USA

**Keywords:** epidemiology, head and neck squamous cell carcinoma, human papillomavirus, personalized medicine, sequencing

## Abstract

While sequencing studies have provided an improved understanding of the genetic landscape of head and neck squamous cell carcinomas (HNSCC), there remains a significant lack of genetic data derived from non-Caucasian cohorts. Additionally, there is wide variation in HNSCC incidence and mortality worldwide both between and within various geographic regions. These epidemiologic differences are in part accounted for by varying exposure to environmental risk factors such as tobacco, alcohol, high risk human papilloma viruses and betel quid. However, inherent genetic factors may also play an important role in this variability. As limited sequencing data is available for many populations, the involvement of unique genetic factors in HNSCC pathogenesis from epidemiologically diverse groups is unknown. Here, we review current knowledge about the epidemiologic, environmental, and genetic variation in HNSCC cohorts globally and discuss future studies necessary to further our understanding of these differences. Long-term, a more complete understanding of the genetic drivers found in diverse HNSCC cohorts may help the development of personalized medicine protocols for patients with rare or complex genetic events.

## INTRODUCTION

Recent next generation sequencing (NGS) studies of head and neck squamous cell carcinomas (HNSCC) have shed light onto the underlying genetic profiles for this aggressive disease [1, 2] and enabled a move towards personalized medicine, in which therapy is guided by tumor genetics. Notably, however, the vast majority of patients sequenced thus far have been restricted to a single epidemiologic population—human papillomavirus (HPV) negative, Caucasian, and high tobacco and/or alcohol use. There has been little information on the genetic profiles in other epidemiologic cohorts; thus, the genomic events driving pathogenesis in these patients remain poorly understood. The rationale to overcome this void is clear and detailed below.

In the US and other high-income countries, personalized medicine approaches are increasingly being applied for many advanced cancers including HNSCC [2–4]. Personalized medicine protocols, such as the National Cancer Institute-Molecular Analysis for Therapy Choice (NCI-MATCH) trial, seek to test molecularly targeted therapies in patients with corresponding mutations [5–7]. However, these protocols often rely on targeted NGS approaches, which are resource intensive and unlikely to be implemented in low- or middle-income countries in the near future. Thus, the idea of targeted and personalized therapy may need to be adjusted in areas where sequencing-based medicine is not yet achievable. One way to do this is to understand the genetic events common to different epidemiologic populations and guide biomarker- based research and medicine towards the most frequent and tractable biomarkers in the region.

Genetic studies comparing ethnic and epidemiologic sub-groups have also been very informative in generally understanding oncogenes and tumor suppressors in cancer. As an example of differential distributions of genetic events based on ethnicity, *TMPRSS2:ETS* gene fusions are found in approximately 50% of prostate cancers in the US, but only 10% of prostate cancers in China. As a result, focused deep sequencing of *TMPRSS2:ETS* gene fusion negative Chinese prostate cancers identified high frequency and previously unrecognized genomic events in alternative pathways [8, 9]. Similarly, we recently performed NGS analysis of an epidemiologically low risk HNSCC (from a young, non-smoker/drinker, HPV- negative patient) with the hypothesis that the tumor would have relatively few mutations compared to a tobacco- related HNSCC. Indeed, our analysis found a potential driver amplification of the tyrosine kinase receptor *FGFR1*. Extending the discovery to The Cancer Genome Atlas (TCGA) HNSCC cohort, we demonstrated that the FGF/FGFR pathway is dysregulated in >30% of HNSCCs and likely represents a previously unrecognized aberration driving disease pathogenesis [10]. Consequently, carefully designed studies focusing on the genetics of under-studied epidemiologic populations can be very informative.

In this review, we will discuss current knowledge of the variations in prevalence, environmental factors, and genetic factors in HNSCC across different regions from around the world. All large (50 or more total patients), available NGS studies in head and neck cancer were included along with other sequencing studies, which were identified primarily using the most recently published PubMed articles after searching for relevant terms. This review also includes discussion of the variation in HNSCC incidence and severity evidenced in black and white American cohorts [11–14]. (In this review, we will use the *New England Journal of Medicine* convention of black as opposed to African American [15]). It is evident from these early studies that different epidemiologic subsets of HNSCC may associate with different tumor genetics and unique outcomes, and thus may be responsive to different targeted therapies.

### HNSCC Rates Globally

**Figure 1:**
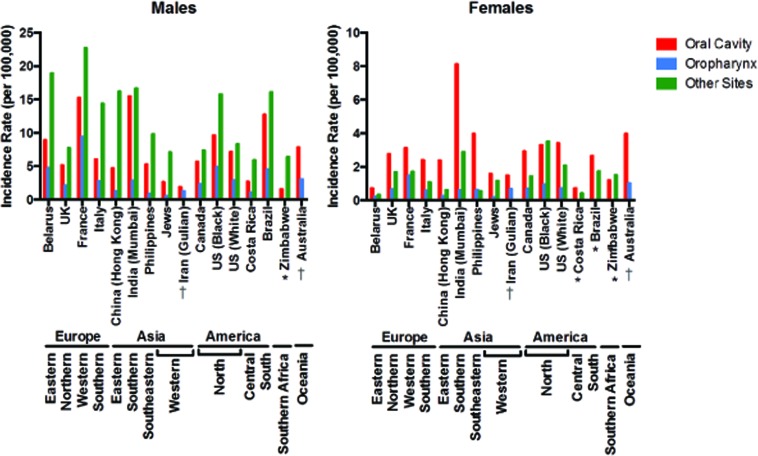
Age-standardized head and neck cancer incidence rates by sex and subsite for various global cohorts Incidence rates per 100,000 for males and females in various global cohorts with cancers of the oral cavity, oropharynx, or other head and neck sites Raw incidences, references, and more detailed descriptions of each study can be found in Table S1. * indicates that incidence for oropharyngeal cancer was not reported. † indicates that incidence for cancer of other sites was not reported.

Historically, different rates of HNSCC have been evidenced in different epidemiologic populations (Figure 1, Table S1). While environmental factors are thought to be a major contributor to this variability, it is unclear if the underlying acquired genetic events are similar across cohorts. Furthermore, the mutational effects of other factors associated with HNSCC globally (most notably high risk HPV strains 16 and 18, but also betel nut in Southeast Asia, nitrosamines in Asia, Epstein-Barr virus (EBV) in Africa and Asia) have been examined in some studies but still require further characterization [16–18]. Here, we will review what is known about HNSCC incidence and mortality in representative countries from around the world.

### Developed Countries: United States, Canada, and Europe

Two thirds of HNSCC cases occur in developed countries, where the use of tobacco and alcohol is prevalent [19]. Odds ratios for developing HNSCC due to tobacco and/or alcohol use are 3-4 times higher in Europe and Latin America, where the use of both substances is more widespread, than in North America [20]. In general, between 1983 and 2002, incidence rates for oral cavity cancers (for which increased risk is particularly noted in smokers) increased in Europe and decreased in the US and Canada [21]. During this time period, incidence of oropharyngeal cancer also increased in eastern and northern Europe. These trends may reflect changes in the proportion of the population using tobacco and/or alcohol.

Tobacco use alone, however, does not account for variation in HNSCC throughout Europe. Based on rates reported by Simard *et al*., HNSCC incidence in all anatomic subsites is somewhat increased in France compared to eastern European countries and is markedly higher in France compared to other European nations (such as the UK and Italy) [21]. Of the representative European countries in Figure 1, however, smoking rates are similar in France, Italy, and the UK. Increased tobacco use, then, cannot completely explain the increased rate of oral cavity cancers between France and other European nations or the higher incidence of cancers of other sites in men from Italy as compared to the UK [21]. While HNSCC in France may be driven somewhat by elevated levels of tobacco use, other factors, including biological differences, may also be crucial for increased prevalence.

### Asia

Increased rates of HNSCC, particularly oral cavity squamous cell carcinoma, in southern and southeastern Asian countries are often attributed to betel quid exposure [22]. Head and neck tumors are one of the most common malignancies in males in some parts of south central Asia. Parkin *et al.* identified the highest incidence of oral cancer in Melanesia (31.5 per 100,000 in men, 21.2 per 100,000 in women) [23]. While nasopharyngeal tumors also have greatest incidence in southeastern Asia, trends in oropharynx cancer vary by specific country [23].

Fewer oral cancer cases are observed in Chinese and Middle Eastern cohorts, where betel quid is used more rarely, as compared to other Asian countries [24]. High rates of laryngeal and other types of HNSCC in China may be due in part to increased tobacco use in this country. Lower incidences of HNSCC at all sites in the Middle East are possible for a variety of reasons, including, but not limited to, the lower use of betel quid, tobacco, and alcohol in this region.

### Africa

There is relatively little data available on HNSCC in African cohorts; nevertheless, tumor epidemiologic differences may exist. A systematic review of the literature since 1990 by Faggons *et al.* found that 7750/8861 (87.5%) patients with HNSCC in sub-Saharan Africa presented with cancer of the oral cavity or oropharynx [25]. Subsite specificity may vary significantly between countries; the nasopharynx was the most common site identified in a review of the literature on head and neck cancer in Nigeria [26], but there were much less frequent reports of tumors of the nasopharynx, nasal cavity, or paranasal sinuses (410/8861, 4.8%), larynx (385/8861, 4.5%), or hypopharynx (66/8861, 0.8%) in sub-Saharan Africa [25]. These differences may be due to difficulties in screening for cancers in these subsites. Consistent with prevalence in the sub-Saharan cohort, oral cavity and oropharyngeal tumors accounted for 27/46 (58.7%) cases in black TCGA patients while larynx cancer was also common (18/46, 39.1%) and hypopharynx tumors were infrequent (1/46, 2.2%).

Within Africa, reports of HNSCC incidence vary widely, from 0.8 per 100,000 in Ghana [27] to 11.1 per 100,000 in South Africa [23]. Tumors of the pharynx and larynx are the second and seventh most common types of cancer seen at the Korle Bu teaching hospital in Ghana, representing 7.4% and 3.5% of all malignancies, respectively [28]. Furthermore, age at tumor presentation in African patients was approximately 20 years younger than in American populations, which may be explained by biological, exposure and/or other differences between these populations [25]. Several epidemiologic factors may contribute to differences in African HNSCC including HIV infection, which has been shown to increase the risk of HNSCC by two to three times in the US [29]. Despite this fact, the role of HIV in African HNSCCs is unclear due to a lack of studies comparing HIV positive and negative patients; anecdotal evidence suggests that HIV positive patients have poorer clinical outcomes but further comparison is necessary [25].

### Variation within Geographic Regions

Variation in HNSCC rates is also observed within different ethnic groups within specific geographic regions [30]. For example, Ho *et al.* considered HNSCC incidence and mortality rates in three Taiwanese tribal groups (Fukkien, Hakkas, and Aboriginal) [31]. Between 1979 and 1997, compared to Fukkien groups, HNSCC mortality rates decreased in Hakka and increased in Aboriginal tribes. Incidence trends between 1979 and 1996 were similar, particularly in Aboriginals with high chewing prevalence for betel quid. While environmental and socioeconomic factors vary between groups, these differences alone may not explain the observed variation in incidence of and mortality from HNSCC. Interestingly, genetic differences have been noted between Fukkiens and Aboriginals in alleles responsible for metabolic activation of carcinogenic nitrosamines [32].

Additionally, significant differences in HNSCC incidence, particularly for larynx cancer, have been noted between black and white Americans. In a study by DeSantis *et al.,* there was a higher incidence of laryngeal cancer in black (10.4/100,000) compared to white males (6.6/100,000); differences were not noted in the incidence of tumors at other HNSCC subsites [11]. Goodwin *et al.* evidenced 15% and 77% increased incidence of oral cavity/pharynx and larynx tumors, respectively, in black as compared to white male Americans. These authors also observed increased incidence of larynx cancer (but not other sites) in black females [12]. The differences in this study were observed to the greatest degree in patients under the age of 65, suggesting that hereditary or early onset factors may be involved. Furthermore, black women displayed higher rates of non-oral cavity, non- oropharyngeal HNSCCs compared to white and other ethnic female cohorts worldwide [21].

HNSCC incidence varies not only by subsite but also by severity and survival rates in groups of black and white American patients. Black patients are more likely to present with late stage, poor prognosis HNSCC than whites [12, 13]. Regardless of cancer site, mortality for black males is on the order of two times higher when compared to that of similar white patients [12]. Furthermore, independent of cancer stage, compared to 63% of whites, only 42% of blacks survive five years following HNSCC diagnosis [11]. While socioeconomic and environmental factors certainly may play a role in this survival difference, the fact that these differences are seen in younger patients suggests a potential role for genetic factors in tumor aggressiveness [14]. Further investigation is necessary to determine these potential hereditary factors and how they may diverge between ethnic cohorts to cause more aggressive disease phenotypes.

### High Risk Human Papillomavirus is Changing HNSCC Epidemiology

Infection with high risk HPV is a major risk factor for head and neck cancers, particularly in the oropharynx [33–40]. Gillison *et al.* reported that HPV positive HNSCCs occurred in 34/60 (56.7%) patients with oropharyngeal cancer but only 10/84 (11.9%) oral cavity, 16/86 (18.6%) larynx, 2/21 (9.5%) hypopharynx, and 0/2 (0%) nasopharynx tumors [36]. Largely due to the increased incidence of the oropharyngeal HNSCC subtype, the recent HPV epidemic is contributing to a rapid change in the epidemiologic distribution of HNSCC globally. To place this in perspective, HPV-positive oropharyngeal squamous cell carcinoma incidence surpassed that of invasive cervical cancer in 2013 [41]. While cervical lesions are often diagnosed early and treated pre-neoplastically to avoid disease progression [42, 43], most oropharyngeal cancer patients present with advanced stage III/IV disease [39]. 70-80% of HPV- positive oropharynx cancers respond to intensive therapy consisting of chemoradiation or surgery in most series [44, 45]. The remaining 20-30% of patients' tumors progress to lethal recurrent or metastatic disease, indicating the need to define biomarkers that will predict the subset of patients that would benefit from more aggressive therapy and will minimize morbidity in less difficult cases. Studies indicating improved prognosis for HPV-positive patients have also suggested that de-escalated chemoradiation therapies, which reduce toxicity and improve quality of life, may be more effective in oropharyngeal, HPV- positive than HPV-negative HNSCCs [46–49]. However, this is not the case for other HNSCC subsites, where rates of HPV infection are typically quite low and might not be an established etiologic factor. Consequently, there are no paradigms for the treatment of oral cavity, laryngeal, hypopharyngeal, or nasopharygeal cancers; additionally, the effects of HPV status on outcomes assessment in these other subsites remains underexplored [50].

In the face of the HPV epidemic, high risk viral infection has been associated with oropharyngeal cancers in studies from across the world. A systematic review by Stein *et al.* compared the prevalence of HPV-positive oropharynx cancer in 23 countries worldwide [51]. Taiwan, Canada, and the Czech Republic had the highest prevalence of HPV-positive oropharyngeal cancer, with much lower HPV burdens in the Netherlands, Brazil, and Spain. Overall, the results of this analysis suggest that HPV-positive tumors may be more common in developed countries. In another systematic review, Mehanna *et al.* identified HPV-positive cancers of the oropharynx in 59.9% of 2550 North American patients, but only 39.7% of 2278 European patients and 32.5% of 568 patients from other regions [52]. An additional cohort of 31/67 (46.2%) Australian patients also displayed high prevalence of HPV-positive oropharyngeal cancer [53]. Chinese patients displayed lower infection rates with 43/207 (20.8%) HPV-positive oropharyngeal tumors and 36/124 (29.4%) HPV-positive tonsil lesions [54]. The prevalence of HPV-positive oropharyngeal cancer was also lower across Africa as compared to many developed countries. 5/125 (4%) South African men were identified with HPV-positive oropharyngeal cancer, and only two of these cases were high risk HPV [55]. Similarly, 0/22 (0%) oropharyngeal cancer and 2/29 (6.9%) oral tongue cancer patients in Mozambique tested positive for HPV infection [56]. In Senegal, only 4/117 (3.4%) HNSCC patients had HPV-related tumors. Of the five patients in this study with oropharyngeal cancer, none tested HPV positive [57].

Of note, the use of numerous testing methodologies to assess a diverse array of HPV variants may introduce inconsistency in HPV detection outcomes. HPV16 accounts for over 90% of HPV-positive HNSCC cases [58] and can be detected by assessing HPV DNA, HPV RNA, viral oncoprotein, cellular protein and/or HPV- specific serum antibody levels [59]. Staining for p16 by immunohistochemistry has historically been used as a surrogate for HPV status, but does not assess for actual HPV infection. Reverse transcriptase (RT)-PCR, PCR- mass spectrometry and in situ hybridization (ISH)-based protocols are also used to detect HPV-positive HNSCC in some cases [60, 61]. Thus, consideration of further studies with controlled testing of the global prevalence of HPV-positive oropharyngeal cancer will be important, particularly in Africa given the discordance between high cervical and low oropharyngeal HPV-positive cancer rates in this region.

**Figure 2:**
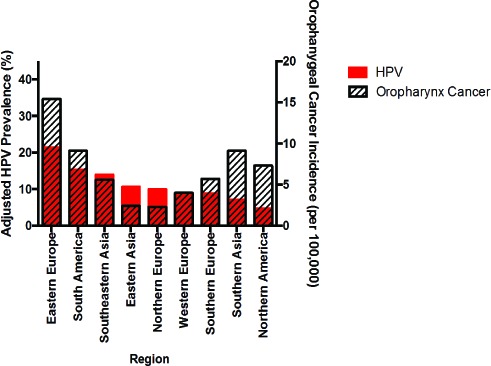
Adjusted cervical HPV infection prevalence among women with normal cytology and oropharyngeal cancer incidence among men by geographic region [66, 69] HPV prevalence includes infection with both low and high risk viral strains and was adjusted based on patient age, year of study, sample type, HPV screening method, and viral strain(s). Oropharyngeal cancer incidence given for a representative country within each region: Slovakia (Eastern Europe), Brazil (South America), Thailand (Southeastern Asia), Japan (Eastern Asia), Denmark (Northern Europe), Netherlands (Western Europe), Spain (Southern Europe), India (Southern Asia), and United States (North America).

While rates of HPV infection worldwide are not fully realized, evidence does indicate that prevalence may be rising globally and may drive increased incidence of HNSCC. Considering the large proportion of the population that is infected with HPV [62], malignant transformation is comparatively rare as HPV infections are usually cleared quickly [63]. In rare cases, however, genomic instability and unrestricted proliferation caused by viral oncogene activity lead to tumorigenesis. Cervical infection with HPV, if not cleared, can lead to precancers in the genital area as well as the head and neck region through sexual contact. Thus, as a high-level surrogate for oropharyngeal cancer prevalence, we can analyze the reports of HPV prevalence in women, noting a wide range of cervical infection rates in cohorts worldwide [64–66]. Based on a meta-analysis of women with normal cytology, Bruni *et al.* estimated that the regionally-adjusted prevalence of high risk HPV infection, as detected by polymerase chain reaction (PCR) or Hybrid Capture 2 (a DNA hybridization assay for detecting HPV strains with a fluorescent readout), in females is 47,271/851,901 (5.0%) worldwide. The prevalence of both low and high-risk HPV strains is 73,019/1,016,719 (11.7%), which varies between rates as high as 75/225 (35.4%) in the Caribbean to those as low as 31/1,435 (1.7%) in Western Asia [66]. Of all viral strains, HPV16 was the most commonly detected in this study but tended to correlate inversely with overall HPV prevalence. In the more limited analysis of data from male patients, an important population given the increased prevalence of HNSCC in this group, a similar overall prevalence (182/1139, 16%) and even more significant amount of variation was observed, particularly when separating low- and high-risk groups [67, 68]. We compared adjusted HPV infection prevalence with oropharyngeal cancer incidence in cohorts worldwide to consider possible associations between these two variables (Figure 2). In a subset of countries, both increased HPV prevalence and oropharyngeal cancer incidence were observed. (For example, women in Eastern Europe have high prevalence of cervical HPV infection (904/4053, 21.4%), and the oropharyngeal cancer incidence in Slovakian men is also elevated (15.4/100,000)). Alternatively, in other regions, decreased HPV prevalence and increased oropharyngeal cancer incidence were noted. (In India, only 1816/23,061 (7.1%) women tested positive for HPV infection, but 9.1/100,000 men develop oropharyngeal tumors [66, 69]). These findings may be explained by regional differences in HPV infection rates; however, developing a more complete understanding of this relationship is additional motivation for controlled HPV testing in global cohorts.

Sexual activity, particularly oral sex with multiple partners, increases HPV-positive cancer risk at all sites. As is the case with other risk factors, sexual practices are widely divergent across populations globally and may be used to at least partially explain differences in the incidence of HPV-positive HNSCCs in various populations. For instance, 78% of American, but only 9% of Indian men reported ever having oral sex, and individuals born after 1960 have more commonly engaged in this activity [70]. Men are also more likely to have multiple partners than women [71–73] and the prevalence of HPV infection was much higher in high-risk populations of males as compared to females with similar numbers of sexual partners [74]. Given these differences, it stands to reason that HPV-positive HNSCC rates will vary widely between cohorts globally.

Variation between HPV-positive head and neck cancer rates has already been observed in whites and blacks in the US and may atleast partially explain racial disparity in survival rates. Settle *et al.* found that differences in the median overall survival (OS) of an American cohort were driven primarily by differences in tumors of the oropharynx (white 69.4 months, black 25.2 months, p = 0.0006) and not by tumors at other sites (white 17.1 months, black 17.5 months, difference n.s.) [75]. In a separate analysis of OS for black and white Americans with HPV-positive or HPV-negative HNSCC, Jiron *et al.* determined that the hazard ratio was greatest for black patients with tumors of the oropharynx but that adjustment for HPV status drastically reduced this ratio to a value close to unity [76]. These results suggest that the poorer prognosis of black patients in the US may be due to the reduced rate of HPV-positive oropharyngeal cancers in this group. Nevertheless, this does not explain differences in laryngeal cancer incidence and survival statistics, and further studies are necessary to more fully evaluate this hypothesis.

### Variation in Genetic Landscape between Epidemiologic Sub-Groups

**Figure 3:**
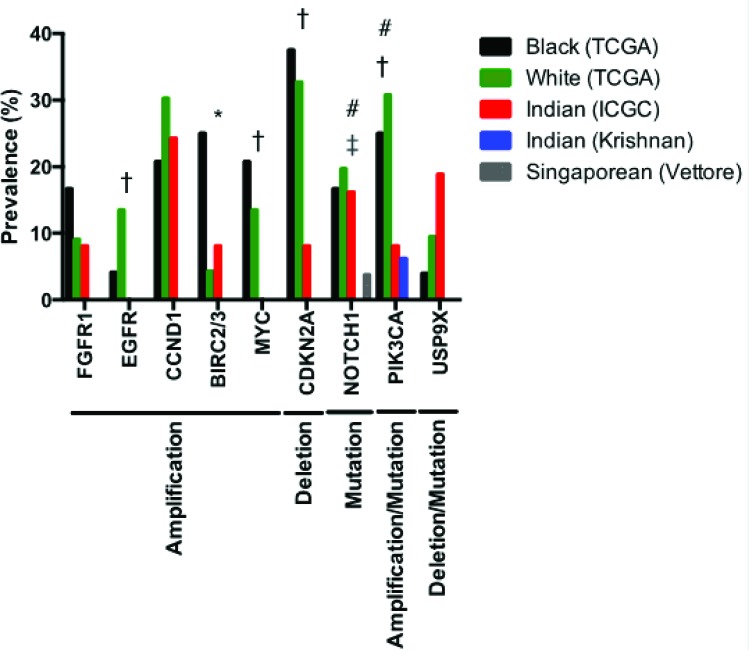
Prevalence of key genetic aberrations in 24 black and 208 white HPV-negative patients (TCGA HNSCC cohort), 37 HPV-negative Indian patients (ICGC HNSCC cohort), 16 Indian HPV-negative patients (Krishnan cohort), and 60 patients from Singapore of unidentified HPV status (Vettore cohort) [1, 16–18] PIK3CA and NOTCH1 mutations only are reported for the Krishnan cohort. NOTCH1 mutation prevalence only is reported for the Vettore cohort. * indicates significant differences between white and black TCGA cohorts. † indicates significant differences between white TCGA and Indian ICGC cohorts. ‡ indicates significant differences between white TCGA and Vettore cohorts. # indicates significant differences between white TCGA and Krishnan cohorts.

Despite the majority of HNSCCs occurring in non- American populations (incidence rate of 60,000 annually in the US vs 490,000 annually in the rest of world), NGS studies have been limited to cohorts of primarily European ancestry and not other ethnic groups or epidemiologic populations. TCGA and the International Cancer Genome Consortium (ICGC) reported sequencing for cohorts of HNSCC patients in the United States and India, respectively [1, 18]. In the TCGA cohort, the majority of these patients were white (242/279, 86.7%), with only 25/279 (9.0%) black. Different mutational profiles were evidenced between black and white HPV-negative patients (Figure 3). For instance, black patients have significantly higher rates of *BIRC2/3* amplification compared to white HNSCC patients in this study (25.0% vs 4.3%, p < 0.001). Although they do not reach statistical significance, blacks also trend toward decreased *EGFR* (4.2% vs 13.5%, p = 0.19) and increased *FGFR1* (16.7% vs 9.1%, p = 0.24) amplification. Other genetic aberrations were similar between these two ethnic groups [77, 78].

ICGC data also noted distinct differences in genomic aberrations in Indian patients with oral cancer of the gingivo-buccal region [18]. When compared to white American HNSCC TCGA patients, Indian patients display significantly lower rates of *EGFR* (p = 0.0365) and *MYC* (p = 0.0365) amplification, *PIK3CA* activation by mutation or amplification (p = 0.0027) and *CDKN2A* deletion (p = 0.0045) (Figure 3). As identified in previous studies of HNSCC, the authors observed frequent copy number alterations or mutations in *TP53, FAT1, CASP8, HRAS* and *NOTCH1* in the Indian HNSCC patients [79]. Interestingly, the ICGC study also identified five genes (*USP9X, MLL4, ARID2, UNC13C* and *TRPM3*) and three pathways (Wnt signaling, dorso-ventral axis formation and axon guidance) previously not associated with HNSCC in TCGA. These events may be specific to this epidemiologic sub-group. For example, mutations in *TRPM3* were identified in 5/50 (10%) Indian ICGC patients, but only 4/208 (1.9%) white and 1/25 (4%) black HPV-negative TCGA patients. *USP9X* was more frequently mutated or deleted in Indian (7/37, 18.9%) than in white (23/208, 11.1%) or black (1/25, 4%) HPV-negative patients [77, 78] although this difference did not reach significance using the Chi-square test with Yate's correction (p = 0.2837). Furthermore, the frequency of copy number alteration or mutation of *FAT1, FAT3,* and *FAT4* were increased in the Indian ICGC cohort [18].

While there have been few other in-depth genomic studies of large ethnic or epidemiologic cohorts, unique mutational profiles are likely to exist in other global populations. Vettore *et al.* recently performed targeted deep sequencing on a cohort of 60 patients treated in Singapore and found that mutation frequencies for *TP53*, *CDKN2A*, and *NOTCH1* were infrequent compared to other studies. While *TP53* and *CDKN2A* represent the first and third most commonly mutated genes in the overall TCGA HNSCC cohort, mutations in these genes, respectively, were present in only 23/60 (38.3%) and 3/60 (5%) of Singaporean patients. Furthermore, *NOTCH1* mutation was identified in significantly fewer of these patients (3/60, 5%) than white HPV-negative TCGA patients (41/208, 14.6%) (p = 0.012). Conversely, *DST*, *RNF213*, *COL6A6 and ZFHSX4* mutations were observed much more commonly in patients in the Singaporean study. Similar studies that could reveal additional trends in other epidemiologic populations, therefore, are clearly warranted.

**Figure 4:**
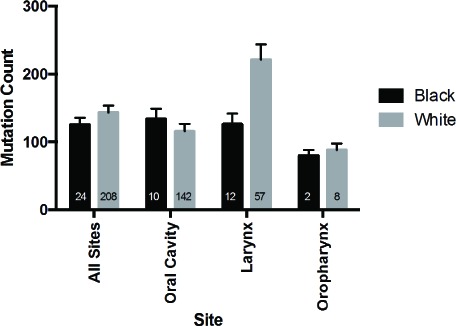
Total mutation load in black and white patients in the TCGA HNSCC cohort Mutation rates were determined based on data from 24 black and 208 white HPV-negative HNSCC patients assessed as part of TCGA.

**Figure 5:**
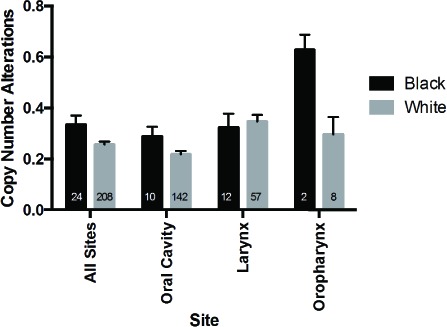
Copy number alterations in black and white patients in the TCGA HNSCC cohort Aberration rates were determined based on data from 24 black and 208 white HPV-negative HNSCC patients assessed as part of TCGA.

The mutational loads were similar between white and black cohorts in TCGA (Figure 4) and Indian patients in ICGC data (mean total number of mutations: 112.79 +/− 19.25) [18]. Copy number alterations, however, were increased in black as compared to white patients (0.3342 vs 0.2560, p = 0.0443) (Figure 5). When subsites were considered individually, trends between ethnic cohorts were apparent for copy number alterations in oropharyngeal tumors (however only 2 black patients compared to 8 white) and were also observed for tumors of the oral cavity (0.2884 vs 0.2174, p = 0.17). These comparisons are limited by the small number of black patients included in this analysis and may be due to sample bias.

### Worldwide rates of established HNSCC molecular events

Currently, *EGFR*, *PIK3CA*, *NOTCH* pathway and *TP53* genes are among the most frequently altered in HNSCC. As these genes have long been associated with HNSCC pathogenesis, several smaller cohort studies have been published assessing the rates of genomic events for these genes in various ethnic and epidemiologic populations. Understanding which populations have unique genetic landscapes may aid in selecting the most informative populations for immediate NGS analysis. Unfortunately, a relatively small number of studies have sequenced HPV-positive HNSCCs in international analyses. Thus, we will separately address worldwide genomic event frequencies in HPV-negative and HPV- positive disease when possible.

### Epidermal Growth Factor Receptor (EGFR)

It has been recognized for nearly 30 years that EGFR is overexpressed in the majority of HNSCC tumors [80]. The effects of EGFR activation in squamous cells may be pleiotropic: not only can changes in receptor signaling affect the Ras-MAPK, PI3K-AKT-PTEN, and/ or phospholipase C pathways, but they may also activate other receptors by ligand-independent dimerization. Consistent with the prevalence of EGFR protein overexpression and demonstrating its importance, EGFR- targeted antibody, cetuximab, is currently the only FDA- approved targeted therapy for HNSCC and has been shown to improve overall survival of patients [81, 82]. A meta-analysis of 37 studies by Keren *et al.* examined EGFR levels in surgically resected primary tumor samples and identified 1948/3346 (57.8%) cases with high protein expression. The majority of patients were from Europe, with some cohorts also from the US and east Asia; overexpression was frequently and consistently noted in Austrian, Spanish, and Dutch cohorts, while it was infrequent in Swedish, French, and Italian populations [83]. High levels of EGFR expression may also be more common in Sudan, where 126/150 (84%) head and neck cancer cases displayed overexpression by IHC analysis [84].

Despite frequent overexpression of *EGFR*, rates of genetic aberration (by amplification or mutation) are relatively low (14.3% in TCGA) [1] and a small number of studies have assessed *EGFR* genomic aberrations in individual populations. For example, 3/41 (7.3%) Korean patients, most of whom had larynx cancer, displayed a mutation in the kinase domain of *EGFR* [85], but similar mutations were much less commonly observed in Caucasian or Spanish patients [86–88]. One TCGA HNSCC patient displayed truncating EGFRvIII mutation. This particular mutation was previously detected by Sok *et al.* in 14/33 (42.4%) HNSCCs along with wild type *EGFR* and was correlated with resistance to targeted EGFR therapy [89]. While mutations of *EGFR* in particular may be somewhat infrequent, this pathway as a whole is often aberrantly expressed. For example, 31/60 (52%) Singaporean patients with tongue cancer displayed mutations in the EGFR pathway (*ADCY8*, *AKT3*, *COL1A1*, *COL1A2*, *EPN1*, *FGFR3*, *FGFR4*, *HRAS*, *HSP90AA1*, *IGF1R*, *ITGAV*, *ITGB3*, *JAK2, JAK3, MTOR*, *PDGFRA, PIK3CD, PIK3CG, PTEN, RASA1, SIPA1,* and *PIK3CA*), and 46/208 (22.1%) of the TCGA cohort displayed amplification of EGFR family members (*EGFR*, *ERBB2-4*, *EGF*, *NRG1-4*, *EREG*, *AREG*, *TFGA*, *BTC*, and *HBEGF)* [1, 17]. An improved understanding of the genetic and/or biochemical mechanisms driving EGFR overexpression in HNSCC will be necessary before extending the assessment of EGFR mechanisms globally. However, as global cohorts are being prioritized for genetic studies, populations such as Swedish and Italian, which have been associated with lower overall rates of EGFR overexpression, may show the largest variation in genomic landscape to those HNSCCs already sequenced. Although *EGFR* copy number, mutation status, and expression level have not been correlated with patient response to EGFR targeted therapies to date, future studies might reveal uses for these characteristics as biomarkers; differences between HNSCC cohorts from various geographic regions could stratify patient groups that might be more responsive to anti-EGFR or other combination treatments.

### Catalytic Subunit of Phosphotidylinositol 3-kinase (PIK3CA)

The PI3K-AKT-PTEN pathway has been identified as the most frequently mutated or amplified oncogenic pathway in HNSCCs in the TCGA cohort [1, 90]. Mutations and/or amplifications in *PIK3CA*, the gene encoding the catalytic subunit of phosphatidylinositol 3-kinase (PI3K), are the most common alterations in this pathway and are observed in 36.9% of the TCGA HNSCC cohort [77, 78]. These aberrations lead to increased cell growth and viability, may drive tumor progression, and are more commonly observed in advanced stage disease as reviewed elsewhere [91, 92]. *PIK3CA* mutation or amplification is also more frequent in HPV-positive tumors, including in the TCGA cohort (56% HPV-positive vs 34% HPV-negative) [1, 90, 93]. Loss of function of *PTEN* also results in failure to “turn off” PI3K signaling and is observed in an additional 10% of HNSCC patients [94].

**Figure 6:**
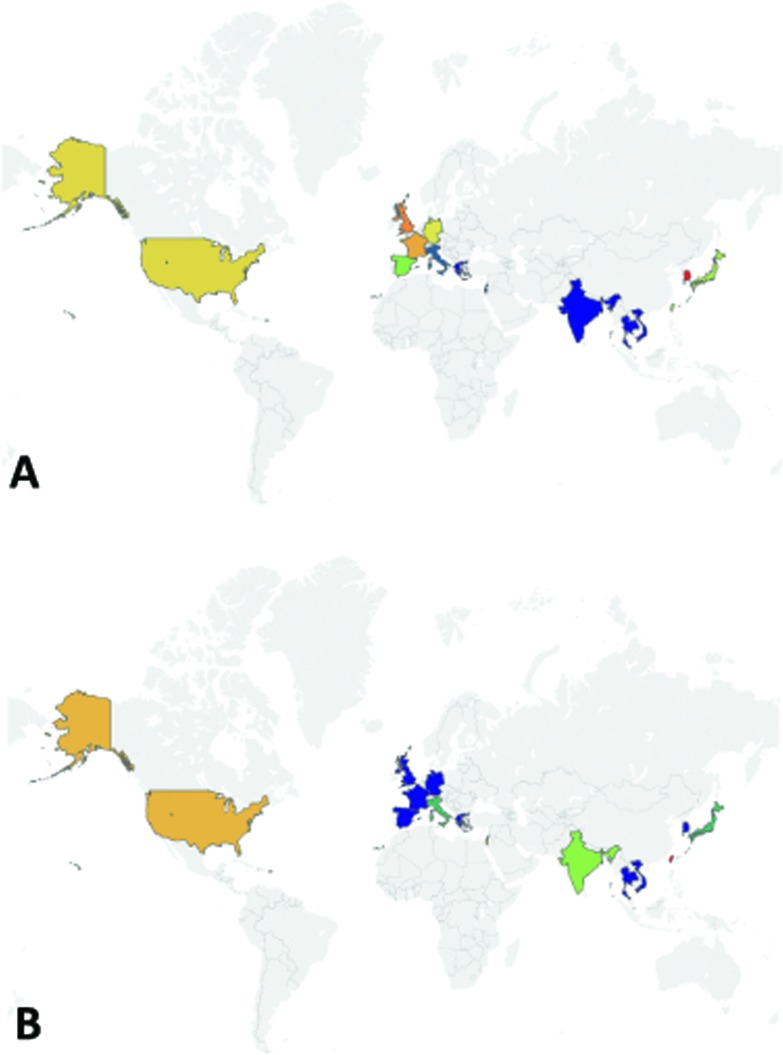
Global variation in frequency of PIK3CA aberration in oral cancer Frequency of PIK3CA amplification (A) and mutation (B) in oral cancer cohorts from countries worldwide. Based on data from Murugan et al. [94] and review of more recent literature, as detailed in Table S2.

Rates of *PIK3CA* aberration have been assessed in various global cohorts [95–100] and are shown in Figure 6. Low frequencies of gene amplification were observed in 3/33 (9%) patients in a German HNSCC cohort as well as 3/115 (2.3%) and 6/50 (12%) individuals in two independent Japanese groups [96, 98, 101]. Alternatively, Redon *et al.* noted 6/9 (66.6%) French HNSCC patients have increased copy number [102]. *PIK3CA* mutation is generally less common than gene amplification. Somatic mutations, commonly including “hotspot” amino acid changes to the kinase (H1047R) or helical (E545K, E542K) domains, occur in 20.8% of the TCGA cohort, which is consistent with rates of ∼10-20% in other studies [95–97]. *PIK3CA* mutation rates greater than 10% were noted in cohorts from Thailand (6/58), India (2/19), and Israel (4/37) [96, 97, 100]. Surprisingly, a complete lack of *PIK3CA* mutations in the helical or kinase domains (exons 9 and 20) were observed in populations of 18 Vietnamese, 33 German, and 86 Greek patients as detected by PCR [97–99]. This may be due to increased activation of *HRAS*, which signals upstream of *PIK3CA*, in these epidemiologic sub-groups [103]. Due to the variation in *PIK3CA* mutation rates between 1/35 (2.9%) and 5/24 (20.8%) in US patient populations [95, 104] and the relatively small number of HNSCC tumor samples that have been sequenced worldwide, additional cohort studies are warranted to further consider potential associations between rates of genetic aberration and patient ethnicity or epidemiologic risk.

### NOTCH pathway genes

In 2011, sequencing-based analysis of HNSCC tumors led to the discovery of inactivating NOTCH pathway alterations as the third most common molecular event in the disease [1, 77, 79, 104]. In fact, mutations in one gene from this pathway, NOTCH1, were observed in ∼15% of samples in addition to less frequent mutations of the NOTCH2 (∼5%) and NOTCH3 (∼4%) genes, with rare copy number alterations reported. Soon after these reports were published, and in contrast to the prevalent loss-of-function mutations, copy number increases and overexpression of the NOTCH ligands JAG1 and JAG2 and the receptor NOTCH3 were found in a small subset of predominantly white HNSCCs [105]. Importantly, the functional role of both the activating and inactivating alterations have yet to be fully characterized. In T lymphoplastic leukemia and other hematologic malignancies, the NOTCH pathway has long been known to have an oncogenic function [106], but the role of alterations in NOTCH1 and other related genes in solid tumors is still the topic of extensive research. NOTCH pathway alterations have been reviewed elsewhere [107], but they are suggested to regulate squamous cell differentiation in multiple model systems of HNSCC. Consistent with this notion, many HNSCCs are characterized by recurrent mutations in the TP63, IRF6 or MED1 genes, which have also been suggested to regulate squamous differentiation, supporting a functional importance of this pathway for HNSCC pathogenesis [79]. Rudy et al. also recently showed that inhibition of WNT signaling via a PORCN inhibitor reduced the metastatic spread of numerous patient-derived HNSCC cell lines in an in vivo chicken chorioallantoic membrane model; this suppression occurred with greater efficacy in cell lines with NOTCH1 deficiency [108].

While the discovery of NOTCH pathway alterations is still relatively new, several studies have assessed the frequencies of molecular events globally. These have demonstrated that 22/51 (43.1%) Chinese oral cavity HNSCC tumors harbored NOTCH1 alterations, with at least half predicted to activate function [109], while only 8/84 (9.5%) Japanese oral cavity HNSCCs had mutations that were all predicted to inactivate NOTCH1 activity [110]. In another cohort of patients from Singapore with tongue cancer (primarily Chinese but also Indian and Malay), NOTCH1 mutations occurred infrequently but alterations in other NOTCH pathway genes (i.e. AR, ARNT, EP300, CREBBP, JAK2, JAK3, NCOA1, NOTCH2, NOTCH3, and PARP1) were common (19/60, 32%) and correlated with disease survival [17]. While much larger cohort studies are needed, the preliminary published data indicates that activating NOTCH pathway alterations may be much more common in Chinese HNSCCs than in patients represented in the Indian ICGC and TCGA HNSCC projects.

Further studies examining the epidemiology of head and neck tumors with NOTCH pathway mutations will better characterize the role of these genetic aberrations in HNSCC. Rettig et al., based on an analysis of 79 tumor samples (primarily from Caucasian patients), reported that strong staining for the transcriptionally active NOTCH1 intracellular domain was more frequently observed in HPV-positive tumors. Mutations in NOTCH1 were more common in HPV-negative cases. In this study there was no difference between these NOTCH pathway alterations when comparing patients based on tobacco and alcohol use [111]. Similar analyses should be performed for other cohorts to confirm these findings and better understand the pathogenesis of the NOTCH wild-type and mutated head and neck cancers.

### The Tumor Suppressor Protein, TP53

The p53 protein functions as a master regulator of the interplay between the cell cycle and apoptosis and is the most frequently deregulated tumor suppressor in HNSCC. In fact, the function and role of p53 in HNSCC have been reviewed extensively due to the high frequency of genetic or biochemical inactivation in the disease [112, 113]. In HPV-negative HNSCC, *TP53* is commonly inactivated by mutation or deletion [114], while the HPV oncoproteins inactivate p53 by biochemical mechanisms in HPV-positive HNSCC. Thus, because *TP53* is usually wild type in HPV-positive HNSCCs, we will restrict the review of genetic events for this gene to oral cavity and larynx HNSCCs, which are historically largely HPV- negative. At these sites, *TP53* mutation often correlates with poorer survival [115] and has been associated with exposure to tobacco or betel quid [114]. Consequently, we may expect to find different rates of *TP53* disruption in different epidemiologic subgroups if the gene is related to these risk factors, or may observe high rates across all populations if inactivation is generally required for squamous pathogenesis.

In the TCGA data set, *TP53* mutation was observed in 129/160 (80.6%) HPV-negative tumors of the oral cavity [1, 77, 78]. In tongue cancer samples from a Singaporean cohort, *TP53* was the most frequently mutated gene (as in TCGA), but was mutated in only 23/60 (38.3%) cases. Relatively low rates of *TP53* mutation are consistent across multiple studies of oral cavity cancer in Asian patients [17, 116, 117]. Cohorts of Icelandic and American never-smokers also displayed lower oral cancer *TP53* mutation rates [118, 119], while rates in Brazil and India were more consistent with those in the HPV-negative TCGA cohort [18, 120, 121]. Many of the patients in the ICGC study had HPV-positive, *TP53* mutant tumors, which is inconsistent with previous studies showing no difference or lower mutation rates in HPV-positive HNSCC patients [122–124]. Other risk factors, such as tobacco use in addition to HPV infection, could contribute to this. Patients with multiple etiological factors are likely to have higher mutation rates and have also been shown to display decreased survival [46]. Further studies of HPV- positive HNSCC in this geographic (Singaporean) or anatomical (oral cavity) subset might better explain these differences.

Global *TP53* mutation rates for oral cavity and larynx HNSCC are summarized in Tables 1 and 2, respectively. In the studies published thus far, *TP53* mutation rates for laryngeal cancers are generally moderately higher than those in oral cavity cancer and also vary by geographic region. For example, in the TCGA cohort, 64/72 (88.9%) HPV-negative patients with laryngeal cancer display *TP53* mutation [1, 77, 78]. Most other countries also have mutation rates of 50% or higher with the exception of China, South Africa, and Argentina [124–126]. Overall, however, these metadata are indicative of a relationship between inactivation and carcinogen exposure as opposed to specific pathogenic requirements.

**Table 1:** *TP53* mutation rates in geographical cohorts with oral cavity cancer

Country	Site	TP53 Mutation Frequency	Reference
US (TCGA)	Oral cavity	129/160 (80.6%)	[77]
Asia	Tongue	23/60 (38.3%)	[17]
Asia	Tongue	7/66 (10.6%)	[116]
Asia	Oral cavity	31/112 (27.7%)	[117]
Taiwan	Oral cavity	26/79 (32.9%)	[115]
India	Gingivo-buccal	31/50 (62%)	[18]
India	Tongue	19/50 (38%)	[16]
US (never-smoker)	Oral cavity	10/61 (16.4%)	[119]
Iceland	Oral cavity	11/52 (21.1%)	[118]
Brazil	Oral cavity	15/30 (15%)	[120]
Brazil	Oral cavity	(40%)	[121]

**Table 2:** *TP53* mutation rates in geographical cohorts with larynx cancer

Country	Site	TP53 Mutation Frequency	Reference
US HPV-negative (TCGA)	Larynx	64/72 (88.9%)	[77]
China	Larynx	22/64 (34.4%)	[124]
Italy	Larynx	62/82 (75.6%)	[128]
Italy	Larynx	36/81 (44.4%)	[129]
Denmark	Larynx (supraglottic)	87/158 (55.1%)	[130]
South Africa	Larynx	11/44 (25%)	[125]
Brazil	Larynx	3/7 (42.9%)	[120]
Brazil	Larynx/hypopharynx	49/58 (69.0%)	[126]
Argentina (Buenos Aires)	Larynx/hypopharynx	2/15 (13.3%)	[126]

Consistent with this observation, several groups have attempted to model the predictive value of individual *TP53* mutations in different epidemiologic populations. For example, Ren *et al.* performed a meta-analysis to assess HNSCC risk and Arg72Pro *TP53* mutation across various tumor sites in Asian and Caucasian cohorts. They found that this mutation was associated with increased risk of nasopharyngeal cancer, but not oral cancer, for homozygous, heterozygous and dominant model mutation comparisons in Caucasian cohorts but only homozygous mutations in Asian patients [127]. Given the frequency and complexity of *TP53* aberration, further studies on the distinct role of this gene in specific epidemiological populations will be critical to developing an improved understanding of HNSCC pathogenesis.

## FUTURE DIRECTIONS

Ultimately, additional sequencing of various epidemiologic sub-groups will need to be performed to understand the distribution of molecular events on a global scale. This work should also assess the correlations of disruptive genomic events with worldwide incidence, mortality, and particularly survival differences, which have not previously been taken into account. There is also a significant void of sequencing data in African populations in particular. Sequencing of these groups may identify both common and unique drivers for HNSCC between various cohorts. Studies of African populations are extremely important due to the high rate of HIV/ AIDS patients in the region. We still have very limited knowledge of the pathogenesis or molecular distribution of HNSCCs in immunocompromised patients such as those who have HIV/AIDS or have undergone organ transplant. Additionally, analysis of cohorts worldwide will be necessary to determine the extent to which other environmental and genetic factors affect the incidence and severity of HNSCC both between and within epidemiologic sub-groups.

HPV-positive oropharyngeal squamous cell carcinoma now displays greater incidence than invasive cervical cancer [41]. While HPV vaccines have the potential to reduce the overall number of tumors caused by this virus, many populations around the world are incompletely vaccinated and it is unknown how many people encountered HPV before having the opportunity to be vaccinated. Thus, given the increasing rates of HPV in the US and abroad as well as wide variation between cohorts, there is a clear and urgent need for both epidemiologic data and sequencing analysis on HPV- positive tumors, especially those that are associated with additional risk factors. These studies have been performed partially, but include only analyses of HNSCC of the tongue or other regions of the oral cavity [17, 18] and not those cases involving more commonly HPV-positive oropharynx tumors. Fortunately, patients in the US with HPV-positive tumors are generally younger and have improved prognosis since HPV-positive tumors are more sensitive to chemoradiation [47]. Despite this fact, some HPV-positive tumors are highly aggressive and rapidly lethal, and there are no established biomarkers that can identify patients that would respond to more aggressive therapy. Future studies are needed to more fully elucidate the specific differences between HPV-positive patients from different geographic regions, cultural backgrounds, and genders using careful genetic analysis in the context of understanding the lethality of each tumor. These efforts are important as they may enable the development of biomarkers for the most aggressive forms of this epidemic subset of HNSCCs.

The heterogeneity of HNSCC has been clearly observed in previous studies and is dependent on tumor genetics and exposure to various risk factors including the use of tobacco, alcohol, and betel quid, HPV infection, and others. This variation is noted between not only between patients but also within individual tumors and between local, nodal, and distant tumor sites. Understanding the broad and underappreciated heterogeneity of HNSCC using comparative genetics will be valuable in establishing personalized medicine protocols first for clinical trials and then for individualized treatment plans. At this point, however, studies have not assessed the genetic heterogeneity found in individual tumors on a large scale. It is possible that different causal or associated factors driving HNSCC pathogenesis will lead to different levels of genetic heterogeneity within a tumor. As recurrence from targeted therapy can arise from individual cells with pre-existing resistant mutations, understanding the degree of heterogeneity in tumors from different epidemiologic subgroups may have a substantial impact on the choice of targeted therapy in each population.

Consequently, further sequencing analysis of patients will likely allow for more effective use of targeted therapies in countries where NGS analysis is readily available. Clinical trials are currently evaluating the response of HNSCC patients to single or dual targeted therapy treatments, such as EGFR (NCT02365662) or PI3K (NCT02145312, NCT02540928) monotherapies or both agents administered in combination (NCT0160231). However, these trials are rarely performed in subsets of patients with lesions that might make them more or less sensitive to particular treatment regimens. Specific genetic or clinicopathological characteristics may correlate with patient outcomes, but this effect will not be realized unless the genetic background of most (or all) of the individuals in the trial is known. One example of a trial that seeks to determine patient response in a subgroup of individuals with molecular enrichment is NCT02649530; this study is intended to evaluate WNT974 as a treatment for HNSCC patients with *NOTCH1* deficient tumors.

Unfortunately, while precision-guided targeted antibodies and small molecule inhibitors display great promise in the future of cancer treatment, their use is currently limited by several disadvantages, including high cost and complex infusion regimens. Since genetic studies are perhaps most feasible in high income countries, understanding the contribution of epidemiological factors to HNSCC development and progression through various genetic pathways may enable treatments to be more effectively selected for patients. For example, understanding the high frequency genetic events in each region may restrict the number of biomarker tests needed to identify tumor drivers, and may enable clinicians to predict whether more or less aggressive therapy is needed based on those markers.

In the future, the hope is that access to healthcare resources and infrastructure will be enhanced globally so that patients have access to the best possible personalized therapy. In the meantime, we must think critically about the cost-benefit of biomarker-guided medicine in different epidemiologic subgroups of HNSCC in order to maximize the return on the high cost of NGS. Analyzing populations with unique epidemiologic and/or biomarker characteristics may be the first step to both enhancing our understanding globally and designing interventional protocols adapted to regional differences in health care resources and tumor genetics. We are in an exciting era of sequencing-guided personalized medicine in the US, and our challenge moving forward is to take the discoveries and lessons from these early personalized medicine trials, incorporate global sequencing information, and improve HNSCC therapy worldwide.

## SUPPLEMENTARY INFORMATION


