# Supplementary material for: Genetic determinants in head and neck squamous cell carcinoma and their influence on global personalized medicine
Source: Genes Cancer. 2016 May;7(5-6):182–200. doi: 10.18632/genesandcancer.110 (PMC4979591; doi:10.18632/genesandcancer.110)
Supplement: Supplementary file 1 [file ganc-07-182-s001.pdf]

Genetic determinants in head and neck squamous cell carcinoma and their influence on global personalized medicine – Michmerhuizen et al

Supplementary Information

Table S1. Age-standardized head and neck cancer incidence rates by sex and subsite for various global cohorts

(21) Incidence rates per 100,000 for adult (age > 15 years) males and females in various global cohorts with cancers of the oral cavity, oropharynx, or other head and neck sites from 1998-2002. Other HNSCC sites include the pyriform sinus, hypopharynx, lip/oral cavity/pharynx not otherwise specified, and larynx.

(131) Incidence rates per 100,000 for adult males and females in Zimbabwe from 1996-2000. Other HNSCC site incidence is the sum of the nasopharyngeal and laryngeal cancer incidence.

(132) Incidence rates per 100,000 for males and females of all ages in Australia with cancer of the lip and oral cavity or oropharynx from 1982-2008.

(133) Incidence rates per 100,000 for males and females of all ages in Guilan, Iran from 2008-2009 with cancers of the lip and oral cavity or pharynx and tonsil from 2008-2009.

***** indicates incidence not available

	**Oral Cavity**		**Oropharynx**		**Other Sites**		
**Country**	***Male***	***Female***	***Male***	***Female***	***Male***	***Female***	**Reference**
Belarus	8.93	0.73	4.75	0.23	18.93	0.34	(21)
UK	5.18	2.75	2.13	0.65	7.74	1.69	(21)
France	15.25	3.12	9.43	1.48	22.69	1.7	(21)
Italy	6.08	2.41	2.8	0.57	14.36	1.08	(21)
China (Hong Kong)	4.72	2.38	1.25	0.26	16.19	0.61	(21)
India (Mumbai)	15.49	8.12	2.82	0.61	16.66	2.89	(21)
Philippines	5.3	3.97	0.93	0.62	9.81	0.55	(21)
Iran (Guilan)	1.91	1.25	1.49	0.67	*	*	(133)
Jews	2.64	1.59	0.57	0.18	7.09	1.16	(21)
Canada	5.7	2.92	2.36	0.7	7.39	1.44	(21)

US (134)	9.65	3.31	4.93	0.95	15.81	3.5	(21)
US (White)	7.16	3.41	2.9	0.73	8.31	2.07	(21)
Costa Rica	2.69	0.73	1.07	*	5.95	0.42	(21)
Brazil	12.73	2.66	4.56	*	16.12	1.73	(21)
Zimbabwe	6.4	1.2	*	*	1.6	1.5	(131)
Australia	7.84	3.06	3.97	1.01	*	*	(132)

Table S2. Raw data for frequency of *PIK3CA* aberration in oral cancer used to generate Figure 6

Bold text indicates amplification of chromosomal locus 3q26 (as opposed to *PIK3CA*)

* indicates that both amplification of *PIK3CA* and chromosomal locus 3q26 were reported (*PIK3CA* amplification rate was used to generate **Figure 6**.)

For countries with more than one value for *PIK3CA* mutation or amplification, an average was used to generate the **Figure 6**.

	**Amplification**				**Mutation**			
Country	**No. amp**	**Total**	**%**	*****	**No. mut**	**Total**	**%**	**Reference**
Japan	3	115	2.6%		3	115	2.6%	(103)
US	14	31	**45.2%**		2	31	6.5%	(103)
Israel					4	37	10.8%	(103)
US					6	74	8.1%	(103)
US					3	120	2.5%	(103)
Taiwan	40	82	48.8%					(103)
US					1	35	2.9%	(103)
Greece					0	86	0%	(103)
Spain	9	24	37.5%					(103)
Vietnam					0	18	0%	(103)
India					2	19	10.5%	(103)
US					5	24	20.8%	(103)
UK	56	68	**82.4%**					(103)
Germany	7	12	**58.3%**					(103)
Germany	3	33	9.1%	*	0	33	0%	(103)
Japan	6	50	12%		2	50	4%	(103)
Thailand	12	58	20.7%		6	58	10.3%	(103)
US					4	38	10.5%	(103)

Taiwan	21	25	**84%**					(103)
Germany	85	280	**30.4%**					(103)
Spain	43	117	36.8%					(103)
UK	34	45	**75.6%**					(103)
US	34	49	**69.4%**					(103)
Germany	4	7	**57.1%**					(103)
US	50	75	**66.7%**					(103)
Germany	26	44	**59.1%**					(103)
France	6	9	66.7%	*				(103)
Japan	29	32	**90.6%**					(103)
Japan	7	11	**63.6%**					(103)
Germany	26	30	**86.7%**					(103)
US	5	10	**50%**					(103)
US	10	13	**76.9%**					(103)
India					2	50	4.0%	(135)
Taiwan					9	50	18%	(136)
Taiwan					58	345	16.8%	(137)
US					45	279	16.1%	(138)
India					2	50	4%	(18)
Italy					2	61	3.3%	(139)
Singapore					3	66	4.5%	(116)
Asia					5	60	8.3%	(17)
Taiwan					11	79	13.9%	(115)
Asia					5	123	4.1%	(117)
US	44	279	15.8%					(138)
Italy	6	64	9.4%					(139)
South Korea	7	7	100%					(140)
India	0	50	0%					(18)
Taiwan	5	123	4.1%					(141)
